# A Review of the Vector Status of North American *Culicoides* (Diptera: Ceratopogonidae) for Bluetongue Virus, Epizootic Hemorrhagic Disease Virus, and Other Arboviruses of Concern

**DOI:** 10.1007/s40475-022-00263-8

**Published:** 2022-09-10

**Authors:** Bethany L. McGregor, Phillip T. Shults, Emily G. McDermott

**Affiliations:** 1 Arthropod‑Borne Animal Diseases Research Unit, Agricultural Research Service, United States Department of Agriculture, Manhattan, KS 66502 USA; 2 Foreign Arthropod-Borne Animal Disease Research Unit, Agricultural Research Service, United States Department of Agriculture, Manhattan, KS 66502 USA; 3 Department of Entomology and Plant Pathology, University of Arkansas, Fayetteville, AR 72701 USA

**Keywords:** Biting midges, Vector competence, Vectorial capacity, Arboviruses

## Abstract

**Purpose of Review:**

*Culicoides* biting midges transmit several pathogens of veterinary importance in North America, but the vector status of many midge species is unresolved. Additionally, the available evidence of vector competence in these species is scattered and variable. The purpose of this review is to summarize current knowledge on confirmed and putative North American *Culicoides* arbovirus vectors.

**Recent Findings:**

While the vector status of *Culicoides* *sonorensis* (EHDV, BTV, VSV) and *Culicoides* *insignis* (BTV) are well established, several other potential vector species have been recently identified. Frequently, these species are implicated based primarily on host-feeding, abundance, and/or detection of arboviruses from field-collected insects, and often lack laboratory infection and transmission data necessary to fully confirm their vector status. Recent genetic studies have also indicated that some wide-ranging species likely represent several cryptic species, further complicating our understanding of their vector status.

**Summary:**

In most cases, laboratory evidence needed to fully understand the vector status of the putative *Culicoides* vectors is absent; however, it appears that several species are likely contributing to the transmission of arboviruses in North America.

## Introduction

*Culicoides* biting midges (Diptera: Ceratopogonidae) are minute biting flies that feed on a wide variety of animals as well as humans [1]. There are currently 151 recognized species in the United States (US) and Canada, several of which have broad geographic ranges [2] and occupy diverse larval habitats, from manure to mangrove swamps [3]. Habitat preferences impact ranges, as well as their potential host associations. Their blood-feeding behavior makes *Culicoides* severe human nuisance pests in some areas, particularly in coastal regions of the US, but their most significant importance comes from their associations with numerous livestock and wildlife pathogens, particularly arboviruses. Globally, *Culicoides* have been implicated in the transmission of over 40 viruses [4]. However, the majority of these associations come from detections in field-collected insects, and do not necessarily imply that midges are the primary, or even tangential, vectors of these pathogens.

In order to confirm that a *Culicoides* species is a competent, natural vector of a pathogen, four criteria established by the World Health Organization (WHO) must be met: (1) the pathogen must be detected in unfed, field-collected insects; (2) insects must be able to become infected with the pathogen by feeding on an infectious host or artificial blood meal; (3) infected insects must be able to successfully transmit the pathogen during feeding; (4) there must be an association in space and time between the suspected vector and infected hosts in the field [5]. Although *Culicoides* have been associated with numerous viruses in the field, using these criteria, they have only been confirmed as the primary vectors of a few, particularly the Orbiviruses, bluetongue virus (BTV), epizootic hemorrhagic disease virus (EHDV), and African horse sickness virus (AHSV), and the Orthobunyaviruses, Oropouche virus (OROV), Akabane virus (AKAV), and Schmallenberg virus (SBV) [6]. *Culicoides* have also been confirmed as vectors of the Vesiculovirus, vesicular stomatitis virus (VSV), though it remains unclear whether they are the primary vectors during outbreaks, or whether other taxa (e.g., Simuliidae spp.) also play an important role [7].

Currently, only BTV and EHDV circulate endemically in the US, with occasional incursions of VSV occurring every 5–10 years. BTV virus and EHDV infect domestic and wild ruminants (e.g., cattle, sheep, and deer), and cause clinically indistinguishable disease. Symptoms in susceptible animals include swelling and cyanosis of the tongue, coronitis of the hooves, hemorrhaging, and death [4]. VSV can infect a number of diverse species, including cattle, swine, horses, and, rarely, humans. Clinical disease is characterized by lesions on the mouth/muzzle and feet. Although VSV does not typically cause mortality, its symptoms can easily be confused with those of foot and mouth disease virus, a highly virulent, exotic pathogen of livestock. Because of this concern, VSV outbreaks are treated as serious potential threats, and can result in economic damage stemming from quarantine and animal movement restrictions [7]. To fully understand the role of *Culicoides* in the ecology and transmission of these pathogens, it is vital to differentiate between vector and non-vector species. Here, we review the current vector incrimination status of North American *Culicoides* species for BTV, EHDV, VSV, and other arboviruses.

## Livestock-Associated Species

### *Culicoidessonorensis*

In the US, only two *Culicoides* species currently meet the WHO criteria for consideration as confirmed BTV and/or EHDV vectors: *Culicoides* *sonorensis* Wirth and Jones and *Culicoides* *insignis* Lutz. Both of these species are primarily associated with livestock production, particularly cattle. *Culicoides* *sonorensis* is the most well-studied Ceratopogonid species globally, though it is endemic only to North America. The ability to colonize this species [8], along with its permissiveness to numerous pathogens and the availability of cell and molecular tools, have made *C.* *sonorensis* the model BTV/EHDV vector. *Culicoides* *insignis* is a Neotropical species, and its range in the US is limited to the extreme southeast, though there is evidence of increasing range expansion [9•].

With some important caveats discussed later in this review, *C.* *sonorensis* is most often reported west of the Mississippi River in close association with livestock. In areas of the southeastern US where this species is considered rare, *C.* *sonorensis* can still be collected from dairy wastewater lagoons, where larvae commonly develop [10, 11]. In the US, natural *C.* *sonorensis* transmission of BTV is well documented; the species is highly competent for BTV infection in the laboratory, frequently tests positive for the pathogen during surveillance efforts, and is found in high abundance in close association with susceptible hosts [12]. *Culicoides* *sonorensis* is also competent for EHDV [13, 14], though its role in the natural transmission of the virus during outbreaks is less clear. EHDV outbreaks are more common in the eastern and Midwestern US [15], where *C.* *sonorensis* is less common, and EHDV-positive *C.* *sonorensis* pools are not often detected during vector surveillance efforts (WHO criterion one; [16•, 17, 18]). The only other virus that *C.* *sonorensis* is thought to naturally transmit in the US is VSV. Elucidation of natural VSV cycles is difficult due to the sporadic nature of outbreaks, and numerous insect species have been implicated in both mechanical and biological transmission [7]. VSV has been detected in field-collected *C.* *sonorensis* from past US outbreaks, including the 2019–2020 outbreak, which affected animals in states from Arkansas to Arizona [19]. Interestingly, there is evidence that VSV may be transmitted transovarially [20] and/or venereally [21] between midges, which has not been shown in Orbiviruses.

Due to how easily *C.* *sonorensis* can be maintained in the laboratory, it has also been used to assess the transmission and replication of other non-US endemic pathogens. Although not present in the currently known distribution of these viruses, *C.* *sonorensis* can successfully develop disseminated infections of AKAV [22], AHSV [23], and SBV [24] after *per* *os* infection. More recently, laboratory studies have confirmed *C.* *sonorensis* competence for the only known *Culicoides*-transmitted human arbovirus, OROV, which is endemic to Central and South America. Eighty-three percent of midges fed an infectious blood meal developed a disseminated infection and > 19% of midges had infectious virus in their saliva, indicating transmission potential [25]. Should these pathogens be introduced into the US, there is a risk that *C.* *sonorensis* would be able to support natural transmission cycles.

In 2018, Möhlmann et al. [26] demonstrated that *C.* *sonorensis* could become infected with Shuni virus, an emerging Orthobunyavirus with zoonotic potential, but that dissemination rates were < 25%, indicating likely low natural transmission potential. Stokes et al. [27] assessed *C.* *sonorensis* vector competence for bovine ephemeral fever virus (BEFV), which is reported to infect cattle in Africa, Asia, Australia, and the Middle East, but found low dissemination rates (~ 1%) in orally infected midges. The authors were also unable to demonstrate transmission between vectors and calves, even when midges were infected via intrathoracic inoculation, a technique that produces nearly 100% infected individuals. It would therefore be unlikely that *C.* *sonorensis* would be able to drive transmission of BEFV in the US were the virus to be introduced. During the 2020–2021 COVID-19 pandemic, there was considerable interest in whether arthropods could biologically transmit SARS-CoV-2 after feeding on a host with a detectible viremia. A laboratory study found that while 85% of pooled *C.* *sonorensis* exposed to the virus in a blood meal did have detectable viral RNA by qRT-PCR in their bodies 10 days post-infection, plaque assays indicated that these positive Ct values did not represent infectious virus [28]. These findings suggest that *C.* *sonorensis* is not involved in SARS-CoV-2 transmission.

### Other Putative Livestock-Associated Vectors

*Culicoides* *sonorensis* is part of a complex of midge species related to *Culicoides* *variipennis* (Coquillett). *Culicoides* *variipennis* complex species are found throughout the US, but only *C.* *sonorensis* is currently considered a competent vector of any known animal pathogens. While these two species do overlap in their distribution, *C.* *sonorensis* is more common in the western US, while *C.* *variipennis* is more common in the east [29, 30••]. In literature published prior to the formal description of *C.* *sonorensis* as a subspecies of *C.* *variipennis* in 1957 (and later as its own species in 2000) [29], authors generally refer only to *C.* *variipennis* when examining vector competence for BTV/EHDV. However, the majority of these studies were most likely conducted using *C.* *sonorensis* sensu stricto. In some cases (e.g., where field-collected insects were tested for pathogens in sympatric areas [13]), it is impossible to know for sure which species was used. There are very few data on the competence of other *C.* *variipennis* complex species. Tabachnick [12] refers to some of his unpublished data on infection rates in field-collected *C.* *variipennis* from New York, New Jersey, and Maryland, and *C.* *occidentalis* from California, where they found low (< 3%) minimum BTV infection rates. More recently, McGregor et al. [19] reported a single VSV + pool of *C.* *variipennis* collected in Kansas during the 2020 outbreak. To date, no laboratory infection experiments using *C.* *variipennis* have been conducted, so the true vector competence of this species for any of the *Culicoides*-transmitted arboviruses is unknown. Other members of the *C.* *variipennis* complex may also be competent vectors. Shults et al. [30••] recently suggested that *Culicoides* *albertensis* (previously synonymized with *C.* *sonorensis*) should be re-elevated to species status (see below), and showed that “*C.* *sonorensis*” collected in Ontario, Canada, preceding a 2015 BTV-13 outbreak [31] were actually *C.* *albertensis*.

The only other confirmed BTV vector endemic to the US is *C.* *insignis*. This species is considered the principal Neotropical BTV vector but has historically been restricted to a relatively small area of the US, in southern and central Florida. Like *C.* *sonorensis* and *C.* *variipennis*, *C.* *insignis* is commonly found in livestock habitats, but utilizes a wider range of development substrates, increasing its potential to transmit pathogens to both livestock and wildlife. Although recent attempts have been made to colonize *C.* *insignis* for laboratory work [32], no lab lines are currently available, making conclusively proving vector competence for any given pathogen difficult.

Due to its ability to transmit BTV, *Culicoides* researchers suspected that *C.* *insignis* may also be competent for EHDV, a phenomenon observed with other *Culicoides* species [33]. Using field-collected adult midges fed an infectious, artificial blood meal, McGregor et al. [34] showed EHDV-2 infection rates in *C.* *insignis* that ranged from 4.3 to 94.4% and transmission rates ranging from 0 to 27.8%. These results confirmed *C.* *insignis* susceptibility to EHDV-2, but also suggested that it is not as efficient of an EHDV vector as *C.* *sonorensis*. Lack of detection of EHDV in *C.* *insignis* pools collected from areas of Florida with active EHDV circulation supports this hypothesis [16•, 34]. Recent detections of *C.* *insignis* in Georgia, Alabama, Mississippi, and Louisiana, outside of its historical US range in Florida, suggest that this species may be undergoing a range expansion [9•]. Based on laboratory and field data, this change may be unlikely to impact EHDV epidemiology, though the potential effects on BTV transmission are unknown.

## Sylvatic and Wildlife-Associated Species

### *Culicoides stellifer *and *C. debilipalpis*

Several additional North American *Culicoides* species have been implicated in arbovirus transmission through the completion of at least one of the WHO vector incrimination criteria [5]. Most commonly, one or both of the field criteria have been completed (Table 1). Due to challenges in working with and colonizing most *Culicoides* species [35], completion of the two vector competence criteria is typically pursued after the two field criteria are met. The species for which the most information is available include *Culicoides* *stellifer* Coquillett and *Culicoides* *debilipalpis* Lutz. In addition, *Culicoides* *venustus* Hoffman, *Culicoides* *obsoletus* Meigen, *Culicoides* *crepuscularis* Malloch, *Culicoides* *paraensis* Goeldi, *Culicoides* *pallidicornis* Kieffer, and *Culicoides* *haematopotus* Malloch are discussed in this section.

**Table 1:** Summary of current WHO vector incrimination critera met for North American *Culicoides* species for arboviral pathogens. The “o” represents evidence for, the “x” evidence against, and the “- “ means untested. In some instances, critera are supported by limited evidence. Please see the text for a full discussion of the vector status of each species

		WHO criteria^1^			
*Culicoides* species	Arbovirus^2^	1	2	3	4
*crepuscularis*	BTV	o	-	-	x
	EHDV	o	-	-	x
*debilipalpis*	BTV	o	o	-	o
	EHDV	x	o	-	o
*haematopotus*	BTV	o	-	-	x
	EHDV	o	-	-	x
*insignis*	BTV	o	o	o	o
	EHDV	x	o	o	o
*obsoletus* (in Europe)	BTV	o	-	-	o
	EHDV	-	-	-	x
	SBV	o	-	-	o
*obsoletus* (in North America)	BTV	x	-	-	o
	EHDV	x	-	-	o
	SBV	-	-	-	x
*occidentalis*	BTV	x	-	-	o
*pallidicornis*	BTV	-	-	-	o
	EHDV	-	-	-	o
*paraensis*	BTV	-	-	-	o
	EHDV	-	-	-	o
	OROV	o	o	o	o
*sonorensis*	AHSV	-	o	-	x
	AKAV	-	o	-	x
	BEFV	-	x	x	x
	BTV	o	o	o	o
	EHDV	o	o	o	o
	OROV	-	o	-	x
	SARS-CoV-2	-	x	-	x
	SBV	-	o	-	x
	SHUV	-	o	-	x
	VSV	o	o	o	o
*stellifer*	BTV	o	-	-	o
	EHDV	o	-	-	o
	WNV	o	-	-	o
	VSV	o	-	-	o
*variipennis*	BTV	x	-	-	o
*venustus*	BTV	o	x	-	o
	EHDV	o	x	-	o

^1^WHO criteria: (1) Pathogen detected in unfed, field-collected insects; (2) insects infected by feeding on an infectious host or blood meal; (3) infected insects able to transmit pathogen during feeding; (4) association in space and time between insect and infected hosts

^2^Arbovirus abbreviations: African horse sickness virus (AHSV), akabane virus (AKAV), bluetongue virus (BTV), bovine ephemeral fever virus (BEFV), epizootic hemorrhagic disease virus (EHDV), Oropouche virus (OROV), Schmallenberg virus (SBV), severe acute respiratory syndrome coronavirus-2 (SARS-CoV-2), Shuni virus (SHUV), vesicular stomatitis virus (VSV), West Nile virus (WNV)

*Culicoides* *stellifer* is a widely distributed species occurring throughout the US, except in Oregon and Washington state [2]. This species has broad larval habitat associations including along stream and pond edges, as well as small puddles, pools, swamps, and springs [3]. Several arboviruses have been detected from field-collected *C.* *stellifer*, including EHDV-2, EHDV-6, and BTV (serotype unspecified) in Florida [16•], BTV-12 in Louisiana [17, 18], VSV-Indiana serotype in Kansas [19], VSV-New Jersey serotype in Colorado [36], and West Nile virus in Louisiana [37]. This small species has proven particularly challenging to work with in the laboratory, yielding few successful laboratory vector competence assays. Early intrathoracic inoculation assays on *C.* *stellifer* for BTV-7 yielded a single positive pool of individuals, but *per* *os* infection was not successfully demonstrated in this study [38].

*Culicoides* *stellifer* is an extremely abundant species throughout much of its range, an observation that has led to its incrimination as an arbovirus vector by several research groups. In Georgia, *C.* *stellifer* was the second most abundant species collected directly from white-tailed deer (16% of collections) and the third most common species in light trap collections (24% of collections) [39]. *Culicoides* *stellifer* has been found in similarly great abundance in several other southeastern US states including Virginia (~ 63% of collections) [40], Florida (~ 10% of collections) [41], and Alabama (~ 37% of collections) [42], as well as in moderate abundance in Colorado [36] and Oklahoma [43]. A direct association between *C.* *stellifer* and ungulate hosts has been shown using drop traps, in which a fine mesh tent is dropped over a host after a period of time to trap any insects feeding on that host, over cattle and sheep in Virginia [44]. More recently, live-animal aspiration results showed that *C.* *stellifer* readily fed on white-tailed deer, and blood meal analysis indicated a close association of *C.* *stellifer* with several ungulate species [45].

Another sylvatic species that has garnered great attention due to its abundance and persistence near susceptible host populations is *C.* *debilipalpis*. The geographic range of this species spans from the southern US to Brazil [2]. *Culicoides* *debilipalpis* larvae occupy moist tree cavities and stumps, making it a common species in forested areas [3]. High abundance, particularly around susceptible animals during EHDV epizootics, has led to the implication of *C.* *debilipalpis* as a vector on several occasions [17, 46, 47]. *Culicoides* *debilipalpis* has been associated with ruminant hosts both through live animal aspirations and blood meal analysis studies [45, 46]. In one laboratory vector competence trial, one pool and one individual of *C.* *debilipalpis* fed a blood meal containing BTV-11 developed an infection after a 14-day incubation [38]. Another assay performed on *C.* *debilipalpis* (as *C.* *lahillei* prior to re-elevation to species status) found that at high infectious titers (5.3–6.0 log_10_ TCID_50_/ml), this species can become infected with EHDV-2, although transmission was not assayed [48]. While EHDV has not yet been recovered from *C.* *debilipalpis* in the field, BTV was detected in pools of specimens collected from Louisiana in 2006, 2012, and from 2016 to 2018 [17, 18, 49].

### Other Putative Sylvatic Vectors

*Culicoides* *venustus* is a robust species found throughout eastern North America [2]. It is associated with diverse larval habitats including stream margins, swamps, puddles, spring seepages, and hoof prints in wet pastures [3, 50]. *Culicoides* *venustus* belongs to the same subgenus as *C.* *insignis* (Hoffmania), and while relatedness to confirmed vectors does not necessarily imply a species is also competent for a pathogen, multiple vector species are common in some subgenera. EHDV was detected from 14 pools of *C.* *venustus* collected during an outbreak of EHDV-2 and EHDV-6 in the Florida panhandle in 2017 [16•], EHDV (serotype unknown) was detected from a single pool of *C.* *venustus* collected during non-outbreak surveillance efforts in Alabama in 2016 [16•], and EHDV and BTV were detected in pools from Louisiana in 2016–2018 [18]. In one laboratory assay, a population of *C.* *venustus* from New York had very low EHDV and BTV infection rates, with just a single positive individual detected for each pathogen [51]. Further testing and surveillance is needed to determine the importance of *C.* *venustus* in BTV/EHDV transmission in the US.

The range of the confirmed Palearctic vector, *C.* *obsoletus*, expands into North America with a broad temperate Nearctic range [2], although Canadian and European populations have been shown to be fairly genetically dissimilar [52]. Little is known about the North American larval habitat for this species, although it is associated with diverse larval habitats in its Palearctic range including manure heaps, marshes, pools, and tree cavities [53]. It is unclear whether these genetic differences reflect population-level differences in vector competence. European populations have been implicated in the transmission of both BTV and SBV [54, 55], while no virus detections have been made from field-collected *C.* *obsoletus* in North America, although this species has been recovered in high abundance directly from, or in the vicinity of, hosts leading to speculation that Nearctic populations may be involved in virus transmission [47].

*Culicoides* *paraensis* ranges from South America to the central US [2]. As larvae, this species is routinely collected from tree hole habitats and other phytotelmata [3, 56]. In South America, *C.* *paraensis* is a confirmed OROV vector [57]. In North America, there have been no positive virus detections in field-collected *C.* *paraensis*, but it has been implicated as a potential EHDV/BTV vector due to the great abundance collected from cattle in Alabama [47] and its status as a confirmed OROV vector.

*Culicoides* *pallidicornis* is another species with both a Palearctic and Nearctic distribution. Within North America, this species is present from Louisiana to Florida, and northwards through Massachusetts and has been found to breed in marshes [2, 3]. *Culicoides* *pallidicornis* tends to be most abundant during the late winter into early spring [58], and has been shown to feed heavily on white-tailed deer during this time, both through blood meal analysis and live animal aspirations. This host association has led to speculation that *C.* *pallidicornis* may be involved in overwintering of BTV or EHDV in some parts of its range [45]. Not much is known about the biology and ecology of the Nearctic populations of this species.

*Culicoides* *haematopotus* has received little attention overall, although virus detection and population abundance indicate that this species may play some role in the transmission of arboviruses. The distribution for this species is large, ranging from southern Canada south through Central America [2], and its larval habitats are diverse, including pond and stream margins, ditches, swamps, and muddy sand bars [3]. No vector competence assays have been published for this species, although a couple of studies have found field evidence of BTV infection in Louisiana [18, 49]. *Culicoides* *haematopotus* has been found in great abundance in areas near susceptible livestock [59]. However, some reports indicate that this species may feed more heavily on birds than mammals [45, 60].

Finally, *C.* *crepuscularis* is an abundant species throughout North America from Canada through Central America [2], and has been reared from numerous larval habitats including pond and stream margins, puddles, roadside ditches, water-filled hoofprints in fields, spring seepages, and sewage effluence fields [3]. *Culicoides* *crepuscularis* has been implicated in BTV and EHDV transmission through virus detection from field-collected individuals in Louisiana [49]. However, *C.* *crepuscularis* may feed primarily on birds [60], and so is thought that it would be unlikely to transmit BTV or EHDV between ruminants.

## Issues Concerning Species Delimitation

Currently, the North American *Culicoides* species are distributed among 13 subgenera and seven species groups, with 10 species remaining unplaced [2]. The phylogenetic relationships between these groups are unknown, and in many cases, there is no evidence that these even represent monophyletic clades. Harrup et al. [61] provides an excellent overview of the current state of taxonomy and phylogenetics within *Culicoides*, and many of the problems highlighted by this work are still relevant. One particular issue raised in this paper was the increasing number of cryptic species being reported, and the potential influence of cryptic species on epidemiological studies [62–64]. The inability to accurately discriminate vector and non-vector species will impact everything from species distribution records to vector competence data. Species delimitation can be especially challenging in *Culicoides* as many vector species belong to complexes of morphologically similar species [65]. Yet, it is vital to identify potential cryptic species to ensure the accuracy of our vector surveillance programs. In addition to molecular evidence, there are several biological indicators that a single species could potentially be multiple species. This includes an expansive distribution, large amounts of morphological or genetic variation, or the ability to utilize various larval habitats [66].

Some *Culicoides* species are known to have fairly expansive distributions, but recent molecular work has attributed some of this to misidentifications due to cryptic diversity. Potential vector species with ranges that span the majority of North America, such as *C.* *crepuscularis*, *C.* *haematopotus*, and *C.* *stellifer* [3], should be further investigated for the presence of cryptic species and/or species complexes to more accurately demonstrate their role in transmission. In addition to having an expansive geographic range [67], significant amounts of morphological and genetic variation have been observed in *C.* *crepuscularis* [68] and genetic differentiation of the COI gene has been reported in *C.* *stellifer* [64]. The potential for multiple cryptic species is especially high in species that have a Holarctic distribution, such as *C.* *obsoletus* [69]. To our knowledge, Barber et al. [52] were the first study to characterize the genetic difference between Nearctic and Palearctic populations of *Culicoides*. Specimens of *C.* *obsoletus* collected in Canada were substantially divergent from individuals collected in Europe. It remains to be seen if these differences represent species-level divergence. Lastly, the larval habitats of the majority of *Culicoides* species remain uncharacterized [70], and this information represents a potentially valuable resource for species delimitation within the genus.

## Changes to the *Culicoides variipennis* Complex

Over the past 60 years, the taxonomic status of *C.* *sonorensis* has been in flux. While *C.* *variipennis* was described in 1901, *C.* *sonorensis* was not recognized as a distinct taxon until it was designated as a subspecies within the *C.* *variipennis* complex by Wirth and Jones [71]. The differentiation of the five subspecies within this complex was based on subtle morphological differences and larval habitat preference. Downes [72] and Wirth and Morris [73] reexamined the morphology of the *C.* *variipennis* subspecies and concluded that these characters were insufficient to reliably differentiate these taxa, leading them to propose further taxonomic rearrangements. Eventually, Holbrook et al. [29] provided morphological and genetic evidence for the species level designation of *C.* *sonorensis* and synonymized two subspecies under it. As a consequence of all of these taxonomic changes, the literature regarding BTV and EHDV in North America can be convoluted, as it is not always apparent which species is actually being studied.

Recent works have provided evidence that both of the subspecies synonymized with *C.* *sonorensis* (*C.* *albertensis* and *C.* *australis*) may represent independent species. The inability to distinguish these species has likely led to the artificial range expansion of *C.* *sonorensis*. Additionally, estimated field seroprevalence rates of several viruses may have been artificially lowered through inaccurately inflated *C.* *sonorensis* population sizes in some collections*.* In a SNP analysis of the *C.* *variipennis* complex, Shults et al. [30••] found molecular evidence of the three previously recognized species plus *C.* *albertensis* and a new, undescribed species from San Diego, CA. Of the 17 populations examined, over half supported more than one species, highlighting the importance of species delimitation in these areas. This work also discovered that *C.* *albertensis*, *C.* *sonorensis*, and *C.* *variipennis* share a single mitochondrial haplogroup, preventing the molecular identification of these species using common mt barcoding genes. The same delimitation patterns found in Shults et al. [30••] were also recovered using a set of newly developed microsatellite markers [74]. One of these markers appears to have species-specific amplification in *C.* *sonorensis*, and could be used to develop a single-tube assay to detect this vector species in pools of *Culicoides* samples. Additionally, morphological and ecological evidence for the species level designation of all the members of the *C.* *variipennis* complex was recently published in Shults (2020) [75].

## Conclusions

**Fig. 1:**
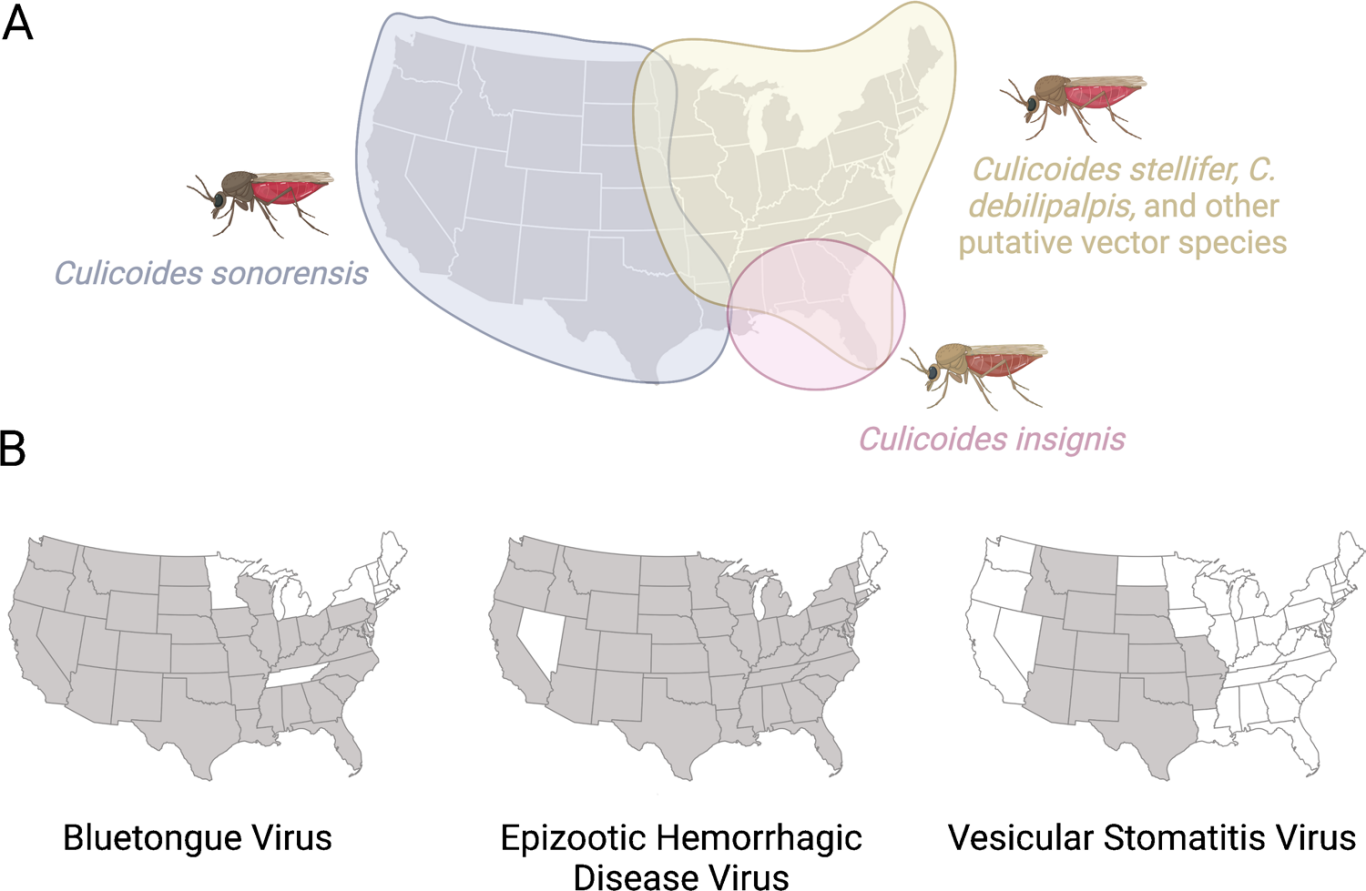
Approximate ranges in which key known and putative *Culicoides* vector species are likely the primary vectors of arboviruses (**A**), and current distribution of bluetongue, epizootic hemorrhagic disease, and vesicular stomatitis viruses (**B**) in the United States (US). Note that the distributions of *C.* *stellifer*, *C.* *debilipalpis*, and other putative *Culicoides* vector species also extend throughout the US, and these species are likely involved in arbovirus transmission throughout the country, particularly to wildlife. *Culicoides* *sonorensis* is also present outside of the depicted range, though it is uncommonly collected in these areas. Virus distribution maps do not indicate prevalence; bluetongue and epizootic hemorrhagic disease virus seroprevalence rates are generally lowest in the northeast and highest in the southeast, but are biased by a lack of reporting in highly endemic areas [15, 76]. The vesicular stomatitis virus map depicts positive states within the past 20 years. Reported virus distribution data come from Ruder et al. [15], ProMED-Mail [77–82], Connecticut Veterinary Medical Diagnostic Laboratory [83], and USDA-ARS [84]

Despite decades of work on the role of *Culicoides* as arbovirus vectors, there remain significant gaps in our understanding of natural arbovirus transmission in North America. Although *C.* *sonorensis* is the only confirmed vector of both BTV and EHDV in the US, it is likely only the principal vector within a limited area of its total potential range (i.e., among livestock in the western US and Canada; Fig. 1). In the far southeastern US, *C.* *insignis* contributes to the spread of BTV. However, in the broader eastern US, BTV, and especially EHDV, are most likely transmitted by one or more sylvatic species. To date, although several species have been identified as putative vectors, none has met all four WHO criteria to confirm their vector status for either virus. Challenges with maintaining many *Culicoides* species in colony have prevented attempts to assess their vector competence under controlled laboratory conditions. Further work on the ecology and behavior of these species is warranted in order to develop effective captive rearing techniques such that these experiments can be completed.

Even within the *C.* *variipennis* complex, considerable questions remain. New genetic approaches have broadened our notion of the diversity of *Culicoides*, and it is becoming clear that a reliance on traditional morphological identification alone may limit our ability to elucidate the role of vector species in arbovirus transmission. That being said, it does appear that *C.* *sonorensis* is highly competent for a number of non-endemic pathogens, and may pose a risk for maintaining them in natural transmission cycles should they be introduced in the US. The epidemiology of *Culicoides*-borne viruses in North America is complex, potentially involving many pathogens and vector species. A multidisciplinary approach, involving ecology, surveillance, genetics, and taxonomy, will be required to better understand these interactions and manage arbovirus transmission to livestock and wildlife.
